# Physician factors affecting patient preferences in selecting a primary care provider: A qualitative research study in Singapore

**DOI:** 10.1371/journal.pone.0298823

**Published:** 2024-03-01

**Authors:** Abigail Ern Jie Lee, Sulaiha Ithinin, Ngiap Chuan Tan

**Affiliations:** 1 SingHealth Polyclinics, Singapore, Singapore; 2 SingHealth-Duke NUS Family Medicine Academic Clinical Programme, Singapore, Singapore; Universitas Indonesia Fakultas Kedokteran, INDONESIA

## Abstract

**Background:**

Care continuity by a dedicated, well-trained primary care physician (PCP) has shown to improve health outcomes of patients with non-communicable diseases (NCDs). In Singapore’s fee-for-service primary care system, patients can choose to consult any PCP in either a public (polyclinic), private (General Practitioners/ GP), or both types of clinics, resulting in potential fragmented care. Decision-making by patients in selecting their preferred PCP remains unclear. This study aims to explore the personal factors influencing the choice of PCP among patients with NCDs in primary care.

**Methods:**

This qualitative research study was conducted in a typical polyclinic. In-depth interviews were conducted on patients with NCDs. Purposive sampling was implemented to enrol patients who had previously consulted PCPs in polyclinics and GP clinics, garnering their perspectives and experiences of care received from both providers. Interviews were audio-recorded, transcribed, and audited. Data was coded and analysed using thematic content analysis to identify emerging themes. The physician-specific factors which influence patients’ decision-making of PCP selection are presented here.

**Results:**

Twenty-one Asian patients aged 38 to 82 years were interviewed. They preferred PCPs with an approachable and genuine demeanour, exhibiting empathy and compassion. They valued the PCPs’ verbal, non-verbal and listening skills. Regarding professional qualities, patients wanted PCPs to demonstrate competency and a patient-centred care approach. Some selected their PCP based on compatible age and gender that they felt comfortable with. Establishing good rapport with their PCP and maintaining continuity of care were deemed as major factors in patients’ PCP selection.

**Conclusion:**

Patients tended to select PCPs based on their personal characteristics, interpersonal skills, professional attributes, demographics, and the physician-patient relationship. PCPs should be aware of these attributes and demonstrate them during their patient interaction. Leveraging on this enables PCPs to build rapport with their patients and maintain care continuity to optimize their health outcomes.

## Introduction

Quality primary healthcare is anchored on four core functions: first contact, coordination, continuity, and comprehensiveness [1]. These qualities are associated with higher quality services, lower costs, less inequality in healthcare, and improved population health [2]. For patients with non-communicable diseases (NCDs) who require long-term care, having a sustained partnership with their physicians through continuity of care is especially important [3]. Numerous studies have highlighted care continuity as an effective strategy to reduce costs and improve health outcomes with associated decreases in long-term mortality [4–6]. Patient preferences often provide primary care physicians (PCPs) with the direction for tailoring interventions in the care of the patients’ NCDs. Healthcare organisations should consequently support PCPs in prioritising continuity of care to optimise patient outcomes, where practice cultures are modified in accordance to changes in patients’ preferences and needs.

Unfortunately, continuity of care has often been overlooked in many healthcare systems that provide episodic, acute care delivered by various providers in different healthcare settings [7]. Such care is centred around short-term encounters with a healthcare provider, where the focus is on addressing the acute medical condition without consideration of a continual care relationship for those with long term medical diseases [8]. Consequently, studies have shown that patients with episodic care experience worse outcomes and incur higher costs, thus reducing the quality of healthcare [9].

Singapore’s healthcare system was originally developed with the intent of providing episodic care, predominantly focusing on treating the acute disease [10, 11]. Within Singapore’s dual fee-for-service primary care system, patient care is delivered by a network of clinics managed by private general practitioners (GPs), or public clinics (polyclinics) where consultations and treatments are subsidised for any local residents [12]. In recent years, subsidised treatment is extended to selected GP clinics, but a ceiling limits the subsidy within a year. Furthermore, Singapore residents presently do not need to follow-up with a regular PCP. A 2022 study reported that only 34.9% of older adults surveyed had a regular family doctor [13]. A primary healthcare system which delivers episodic care will be insufficient to meet the rising prevalence of NCDs as the inconsistent care being received by patients will increase the associated disease burden [14, 15].

Singapore’s healthcare system has recently undergone a major change to improve population health. One of the highlights of the new national “Healthier SG” programme addresses continuity of care, which nudges Singapore residents to commit to one family doctor. Residents are encouraged to select and enrol with a PCP who will be the first point-of-contact to comprehensively manage the residents’ health [16]. It is essential for the primary healthcare providers and policymakers to appreciate the decision-making process of patients in selecting their PCPs, so that they can effectively implement and scale the Healthier SG programme, thereby enhancing the health outcomes of the local population. This study aims to explore the personal factors influencing the choice of PCP among patients with NCDs in primary care. Understanding such factors can assist the PCPs to mould their care-delivery behaviour, and sharpen their communication skills to partner their patients over their long-term management of their NCDs.

## Methods

### Theoretical framework

This study used a qualitative descriptive research approach to explore the personal attributes which influenced patients in choosing their PCPs [17]. The “Generalist Wheel of Knowledge, Understanding and Inquiry” was used as the theoretical framework in this study for clarity and ease of understanding of the inter-related factors [18]. This paper presents the physician factors which influence patients in selecting their PCPs for NCD management in primary care.

### Setting

The study was conducted in one of the twenty-three public polyclinics located in the eastern part of Singapore which managed approximately a thousand patients in a single workday. The demographic profiles of the patients in the study site were similar to that of the general Singapore population. The polyclinic was largely funded by the government and provided subsidised care for patients. Patients who visited the clinic were of all age groups and attendances were for any medical condition, or for preventive health measures. The interviews were carried out from June to December 2022.

### Study team and reflexivity

The study team consisted of three PCPs (AEJL, SI, NCT) and a senior staff nurse (SJT) who practiced in the primary care setting and were involved in patient care. Four medical students were involved in gathering the qualitative data. Prior to study commencement, the investigators did not have any relationship established with participants.

### Participants and sampling

Purposive sampling was implemented to enrol patients who had previously consulted both polyclinic and GP clinics, to capture a wider spectrum of views. Investigators approached potential participants at the study site for face-to-face recruitment. Recruitment was done between 7 June 2022 to 26 November 2022; twenty-three patients fit recruitment criteria and gave consent to participate in the study. The inclusion criteria included multi-ethnic Asian patients of both genders, aged 21 years old and above, with at least one pre-existing NCD. The NCD is defined by the Ministry of Health Chronic Disease Management Programme and documented as diagnosis codes in their electronic medical records [19]. Patients were on regular follow-up for their NCD, and were conversant in English, Chinese or Malay. Those who did not have mental capacity to give informed consent were excluded. Patients were given the Participation Information Sheet and any queries on the research study were addressed by the study investigators.

### Topic guide

The investigators developed a topic guide to interview participants for this study, based on literature and mutual deliberation (see S1 File). It focused on patients’ previous interactions with their healthcare providers, reasons for their choice of healthcare provider and their perception of an ideal PCP. The use of open-ended questions allowed patients to contribute rich information that could be subsequently analysed.

### Data collection

A total of twenty-three patients satisfied eligibility criteria and provided written informed consent to enrol into the study. Subsequently two of them dropped out: one declined interview and one was uncontactable. A questionnaire was administered to collect baseline demographics of each of the remaining twenty-one patients. They were informed of the details of their scheduled interviews and were also given the topic guide to look through prior to the interview. All study materials were translated into Malay and Chinese by professional translators.

The interviews were conducted either face-to-face in a quiet room at the study site, or through video call. The interviews were moderated by three female study team investigators (AEJL, SI, or SJT). Prior to the interview, patients were reassured of confidentiality of their identities. In-depth interviews (IDIs) were conducted in English, Malay or Chinese based on participants’ preferred language. The interviewees were anonymized and addressed by their study identification. Field notes were taken by study investigators during the interviews. No non-participants were present. The interviews were audio-recorded and transcribed verbatim by engaged independent professional transcribers. Interviews conducted in Malay or Chinese were professionally translated into English for subsequent data coding and analysis. The transcribed texts were audited independently by study investigators to ensure consistency. The lead author (AL) who analysed the Chinese interviews uses Chinese as a mother tongue and communicates daily in Chinese fluently. Similarly, the co-author (SI) who analysed the Malay interviews studied Malay as a mother tongue language and communicates daily in Malay fluently. The duration of each IDI was about 20–40 minutes. Patients were given a token of appreciation for their participation. Each participant was interviewed once and no repeat interviews were carried out.

### Data coding and analysis

After familiarizing with the transcripts from the first two IDIs, investigators AEJL and SI independently coded the data. A list of codes was created and assembled to form a preliminary coding framework with the aid of NVivo version 12 software. Any coding variations were resolved by deliberation between AEJL, SI and NCT to finalize the coding framework. Subsequently, AEJL and SI used this framework to code the other transcripts. New codes were subsequently added as an iterative process when more interviews were coded. The new codes were discussed and agreed between investigators subsequently.

The investigators reviewed the codes and leveraged on an iterative process to identify emergent themes. The interviews were concluded when no new theme emerged from the thematic analysis. Data saturation was reached after 18 IDIs. Three more patients were interviewed without any new theme.

### Ethics approval and consent to participate

The study received ethics approval from the SingHealth Centralized Institutional Review Board (reference number 2022/2071), in compliance with the Helsinki Declaration. Written patient consent was obtained.

All methods were performed in accordance with the consolidated criteria for reporting qualitative studies (COREQ) checklist.

## Results

A total of twenty-one patients between 38 to 82 years from all major ethnicities in Singapore were interviewed. Their profiles are described in Table 1.

**Table 1:** Demographic profiles of study patients.

Demographic	Patients (n = 21)	Percentage (%)
**Gender**		
Male	12	57
Female	9	43
**Age (years)**		
21–40	1	5
41–60	6	28
61–80	13	62
> 80	1	5
**Ethnicity**		
Chinese	18	86
Malay	2	9
Indian	1	5
**Highest education**		
Primary	3	14
Secondary	10	48
Post-secondary or Diploma	4	19
University degree and above	4	19
**Employment**		
Homemaker	3	14
Employed	8	38
Retired	10	48
**Chronic conditions**		
Anxiety	1	5
Asthma	2	9
Benign Prostatic Hyperplasia	1	5
Chronic Kidney Disease	1	5
Diabetes Mellitus	2	9
Hyperlipidaemia	6	28
Hypertension	10	48
Ischemic Heart Disease	4	19
Osteoarthritis	2	9

### Principal findings

**Fig 1:**
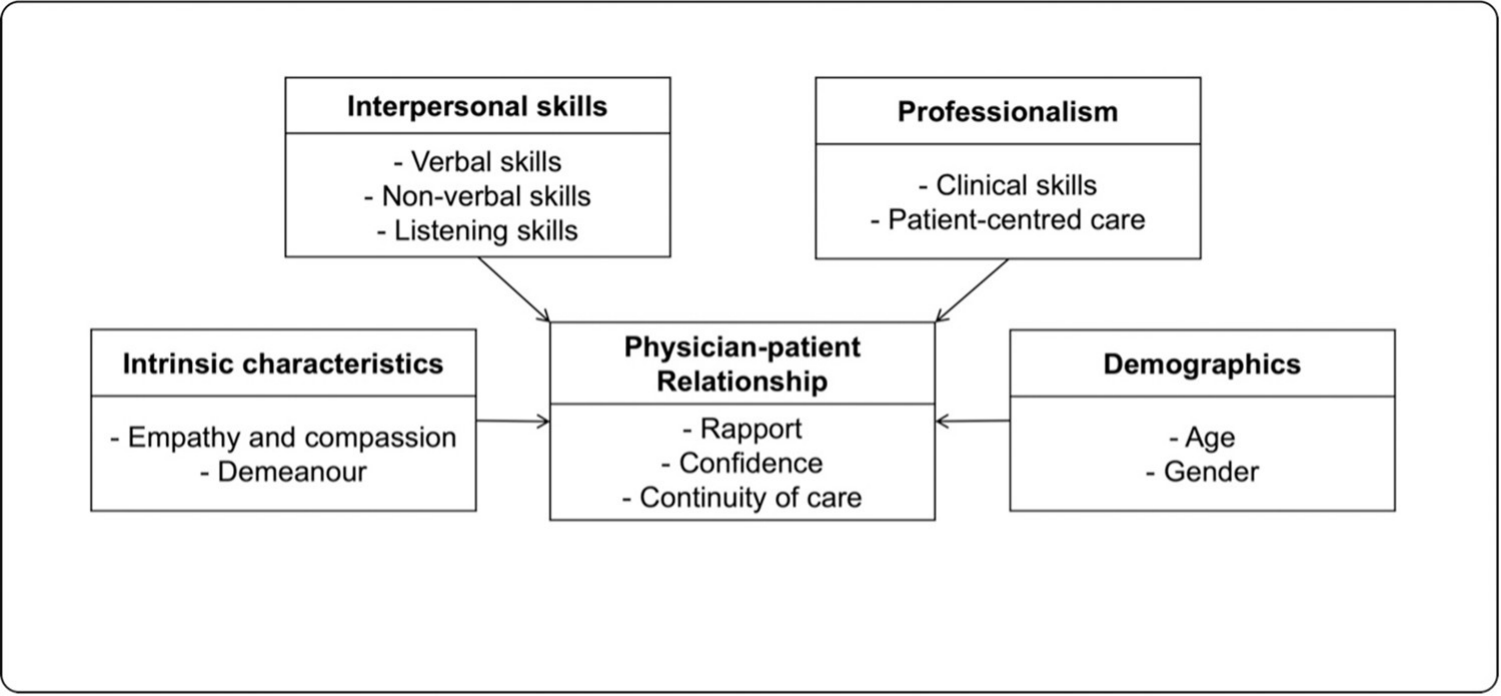
Physician factors influencing the choice of primary care physicians among patients with non-communicable disease in primary care.

The investigators categorized the results according to the following PCP factors: intrinsic characteristics, interpersonal skills, professional attributes, demographics, and the physician-patient relationship. These fall under the “clinician” domain of the Generalist Wheel theoretical framework and how it will affect the relationship between the “clinician” and “patient” domains. The findings are summarised and presented in Fig 1.

#### PCP intrinsic characteristics

*Empathy and compassion*. Patients preferred consulting a PCP who would be able to demonstrate empathy and compassion. They felt that this would allow them to have more confidence in the PCP.

*“So once [the doctor] knows the patient, understands the patient’s experience, the feeling of being in her shoe or his shoe, then he or she will have confidence in the doctor, you see.” S11*

*“To be a doctor, you got to be very compassionate. You got to be very, you know, sympathetic towards the patient, caring, you know, find out more. [The doctor’s] purpose here is to help [patients] to get well, you know, not to get worse. So they must have that type of mentality, you see.” S02*

*PCP’s demeanour*. Patients highlighted various aspects of what they would appreciate in a PCP’s demeanour. They valued PCPs who were approachable and showed consideration and care.

*“She is very friendly, very caring. That’s why I go and see her.” S22*

*“Someone who I can be comfortable with, yeah. … I think somehow through our age, when we are talking to somebody, we can tell whether this person is sincere or not, whether he really is a caring person or not.” S06*

Patients also looked for dedicated, genuine, and sincere PCPs that would be patient with them.

*“You can sense that the doctor is more genuine and concerned. Not that you can talk to the doctor about anything personal, but more so about the medicine and the health-related issues. To get some more information and things like that.” S05*

*“I’d like to have doctor who doesn’t feel so rushed. Who can just, you know, spend some time understanding my problem and things like that, yeah.” S09*

One patient related an experience she had with seeing a pleasant physician who gave her a sense of satisfaction after the consult.

*“She was so helpful, she was very pleasant to me, she was really concerned, and she really spoke like a genuine doctor, and from then on, I see her most of the time now… I stuck to the polyclinic, which I’m always very happy with, you know.” S14*

#### Interpersonal skills

Patients highlighted various aspects of PCP’s interpersonal skills that they felt were important. These were divided into verbal, non-verbal, and listening skills.

*Verbal skills*. Clarity in explanation was something that patients looked out for in their physicians. They preferred physicians who were able to give appropriate advice to them that was easily understood. They also appreciated when communication was two-way.

*“I think a good doctor… has to be able to maintain rapport with the patient, then be concerned about the patient. Be able to give the patient certain explanation of why the prescription is being made. Or why a certain lab test is needed. Basically, is to like, try to not just function as a doctor; it has to go beyond that.” S14*

*“There’re certain things that… Maybe to us are a great problem. But to the doctors, they have seen many patients and they felt that there’s no need to worry about this, you know. So, the doctor can explain it clearly. Then we can understand you know. … I think the doctor has to be very clear in delivering what he wants to tell us.” S13*

*Non-verbal skills*. PCPs’ mannerisms were also important to patients. Apart from the content itself, patients also valued the tone in which PCPs spoke to them, which would convey to the patients what the PCPs’ moods were.

*“The kind of doctor that I’m comfortable with is like, uh, she’s very quick to pick on when I tell her what’s wrong with me… A doctor who listens and a doctor who’s able to quickly, you know, give me the assurance that, okay, don’t worry, you’re in safe hands, you know, yeah.” S08 “The manner, the way doctors phrase the questions, the patient will sense it you know. As a patient we can sense it, whether this doctor is friendly.” S19*

Many patients also highlighted how it was important for PCPs to be mindful of their body language.

*Doctors that are friendly; eye contact is very important. Doctors that talk to the patient in a nice soft manner, … you know, reassure them, connect with them, you know, that’s very very important. I just watch their body language. Yeah, very very important.” S14*

One patient related a negative experience he had with a PCP who did not have good communication skills.

*“As patients, you know, we always want to know more about what we are coming here for. Maybe if it’s a regular doctor, more or less, you may get to know each other. Then maybe you can ask a few questions more. Because frankly speaking, when we visit, some of the doctors they don’t even look at us. …. [One] example, I was waiting outside. Then the appointment was already overdue. I went into the room; he didn’t even look at me. Then he looked at his handphone. Then suddenly when looking at the monitor he then asked me, ‘How are you,’ or something like this. I was in shock you know; I did not know whether he was talking to me or talking to whoever it was. You know, the first meet-up and the perception of this fella is like that, and he is not so friendly already. Of course, I did not want to… I mean we are all human beings. I did not want to make a big fuss out of it. So, he look see, look see. ‘Okay lah no problem, I discharge you. So, you do not have to come in more.’ Like that. So, I went back, of course I didn’t know what happened. Only thing is that I am okay that’s all.” S19*

*Listening skills*. Patients preferred PCPs who were attentive to them when they were speaking. They appreciated when PCPs listened to them wholeheartedly.

*“Ideal doctor is one (who) listens. Don’t be so much on the keyboard”. S13*

*“You can really sense whether a doctor is listening to you wholeheartedly or listening to you for the sake of going through the motions. And she was really quite genuine, listening to you.” S05*

Overall, patients valued PCPs who were able to demonstrate all three aspects of interpersonal skills.

*“My ideal doctor is a doctor who listens, and he will not look impatient, will not look angry, because when you are sick you do not want someone who’s angry or irritated at you. You want someone to be kinder to you and explain. And make you understand what it is that you’re facing, why is it dangerous or not, what you should do. Make them understand. Because if you don’t explain properly, we have to go around asking other people, you know. What does it mean when he said this, he said that he must be a person approachable enough for us to say I don’t understand, can you explain more. Then you feel better.” S04*

#### Professionalism

*Clinical skills*. Patients wanted to see PCPs who could handle their medical conditions. This was measured in the form of having appropriate qualifications as well as adequate experience. They viewed this as part of the PCPs’ professionalism.

*“I would prefer if they were better qualified. Because they will know more, and they will be able to explain to me.” S12*

*“Because when [doctors] study more, [they] have the experience, [they] have experienced more medical conditions and patients explaining it to them. So [they’re] probably more experienced, so it will be better.” S07*

*Patient-centred care*. Patients appreciated when PCPs were able to provide patient-centred care. This involved holistic care of the patient, even if PCPs had to go beyond their call of duty.

*“The doctors there really take care of you, and their care, is sort of holistic in the sense that they check, not just your BP and your cholesterol levels but also any other problems that you might have.” S04*

*“I would prefer a doctor that is more detailed and more thorough. And ask questions rather than the superficial, you know, two or three questions and then that’s it.” S01*

Another aspect involves shared decision-making. Patients valued playing an active role in their disease management. They felt that making informed decisions regarding their medical conditions gave them a sense of empowerment and responsibility over their own health.

*“The doctor has to be very clear in delivering what he wants to tell us. Yes, the pros and the cons you know. And then of course the choice is up to us and then the doctor also can tell us from his experience, you know. That this one will work for more patients you know. So then after that you know it’s up to us to choose, yeah?” S13*

*“I have been contributing to the management of my condition… and that kind of gave me a kind of empowerment also, patient’s empowerment.… I know what’s happening to me and I also hold responsible for what’s happening to me.” S23*

Finally, some patients were also wary of PCPs whose decision-making was influenced by other factors apart their patients’ well-being, that could result in overprescribing of medications and elevation of consultation fees. The Singapore government introduced the Pioneer Generation Package for Singaporeans born on or before 31 December 1949. These pioneer Singaporeans have added healthcare benefits, among which includes outpatient care subsidies that can be used in both polyclinics and GP clinics under the Community Health Assist Scheme. Thus, pioneers seeing GP clinics would only need to pay a minimum amount regardless of the number and type of medications prescribed by the GP.

*“Because I sometimes worry that doctors may overprescribe. … I brought [my mother] to the [doctor’s], so the [doctor], you know, kept on saying, ‘She’s a pioneer, she’s a pioneer’, so I think because of that she has a lot of subsidies, so certain medications which I felt that were not necessary, were given to her. Then when I questioned the doctor, the doctor couldn’t really answer my question.” S13*

#### Demographics

*Age*. There were mixed opinions about the age of the PCP. Some patients felt that this was important, and they would prefer to see an older PCP who would be able to communicate better and they would have more confidence in.

*“Some doctors are too young; you have no confidence. That is what I feel.” S22*

One patient initially felt that it would be better to see an older PCP who would be more experienced, but through her encounter with younger PCPs, she appreciated their demeanour and changed her perspective.

*“I used to think that the older doctors will be better because they are longer in service, they are more experienced. But I think I’ve been proven wrong because sometimes doctors who have been around on time, they’re tired, you know. They get quite impatient, but I’ve seen young doctors. They’re very cheerful, and they sort of pass it to you, you know? They take it easy, and they give you ideas, what to do. Sometimes these young people have new ideas which are worth listening to and doing. It doesn’t mean that you must take only the older ones, but these young ones they have good ideas. And because of their cheerfulness and you know, you feel okay. I thought that you must go to an older doctor. Now I don’t.” S04*

Another patient felt that the PCP’s age did not accurately reflect his or her experience and would still be willing to see a younger PCP who could provide appropriate management.

*“I wouldn’t say older, I would prefer to use more experienced, so it’s like, for chronic especially, the more experienced ones would be better… I mean for me I would look at the way they manage you. Like the medication how they handle… can see that they are inexperienced, as in they’re less experienced. … So, I will look at experience only, not by age. So even those young physicians also can do a good job.” S23*

*Gender*. Some patients preferred to see a PCP of the same gender for ease of communication.

*“I (male participant) personally prefer male doctors, because I have other problems, you know, so it’s more convenient.” S01*

*“Female [patients] of course prefer female [doctors]; it’s easier to communicate and understand.” S07*

However, there were also other participants who did not have any issue with the PCP’s gender.

*“Gender is not an issue of course.” S23*

#### Physician-patient relationship

*Rapport*. Patients valued having good rapport with the PCPs they were seeing. Having a close and harmonious relationship with their PCPs allowed them to feel comfortable during their clinic consults.

*“I firmly believe that [between] a doctor and a patient there must be a relationship, a connection. I think “connection” is the right word to use; that we are able to connect with them and they are friendly enough, that they are sort of frank enough. I mean there is this connecting factor you know. … So I think that connection and comfort level is important.” S06*

*“To me it was very important that I find a doctor that I’m comfortable with. Yeah, you know, because you know, sometimes you’re just so used to the doctor, and you don’t have to think much and you just go. And you know that you can just leave everything in their hands, you know what I mean, yeah, so that to me is a big factor. To be able to just leave everything, I mean, considering what I went through when the [previous] doctor couldn’t quite diagnose my condition, you know? That caused me a lot of anxiety. Yeah. So therefore, to me it’s important that I can find a doctor that I’m comfortable with.” S08*

*Confidence*. Patients wanted to see a PCP that they had confidence in. This was in part measured in terms of the PCPs’ professional attributes.

*“If the doctor… gives you the sense of security and also confidence that they know what you’re talking about, like I said earlier on that they understood you what you say, then you feel more… like it’s worth it you know.” S02*

*“Overall, I’m still confident of the polyclinic. I mean the standard of delivery; I mean in terms of the knowledge and the professionalism that they are delivering. I am still confident that they are doing a good job.” S06*

*Continuity of care*. Patients chose to follow-up with same PCP so that they would not have to repeat their medical history to a different doctor during subsequent consults. Seeing the same PCP would also allow the PCP to be more familiar with the patients’ care.

*“I prefer to see the same doctor, at least they would know your issues from the beginning to the end … they know your medical history, information about your cholesterol, what medications you are taking.” S07*

*“For chronic conditions, for me I personally feel more comfortable you know, consulting the same physician. So, because they know us better, they will know our progress better, because if someone comes and substitutes or you know, cover for another doctor, of course [the doctor] will see, read the previous case notes of mine, but not like previous previous’ previous one right. I am very sure that, I’m sure that the most they would have seen the previous case notes. The previous visit that we had. Yeah, so that’s the reason.” S23*

Patients also valued the continuity of care to strengthen the rapport between themselves and the PCPs.

*“I would want to stick to the same doctor because he will know my history and know my detail better, even though my record is in the system. … We make jokes around also; he is very caring and… just like a friend to me. … I find I open up and he opens up to hear my issues and everything more easily; I think that there’s a rapport which is very good.” S17*

*“This idea of having a family physician, I think it’s very good, its brilliant to be very honest. I think it’s good. Like I said, to give people a peace of mind, comfort-level. But that is provided … they are on the same sort of frequency and the comfort level most importantly.” S06*

## Discussion

The results demonstrate the complex multi-faceted decision-making process among patients in selecting their PCPs based on the physician factors alone. They considered the PCPs’ characteristics, interpersonal skills, professionalism, and demographics when choosing their PCP. Most patients considered a sound physician-patient relationship to be fundamental in strengthening their trust of their PCP. Such a trusting relationship would pave their continuity of care by the same provider.

Patients desired to be shown empathy and compassion by their PCPs. They described their ideal PCP to be friendly, caring, helpful, sincere, and pleasant. Providing such care would require PCPs to have the ability to comprehend patients’ experiences and effectively convey that understanding, aiding patients in managing challenging emotional states [20]. These desirable attributes have been demonstrated in other studies, where patients appreciate PCPs who would manage not only their NCDs but care for them holistically as well [21–23].

Another attribute desired by patients was PCPs’ good interpersonal skills, which is crucial in improving the physician-patient relationship and providing quality care [24]. Effective physician-patient communication improves patients’ health literacy, deepens their understanding of the disease and treatment options, and identifies their ideas, concerns, and expectations. This facilitates shared decision making in formulating a mutually acceptable management plan between the patient and PCP [21, 25]. Patients having good communication with physicians are also more likely to have improved patient satisfaction that can contribute to their fidelity to their management plan [26].

Patients also considered physicians’ professionalism when selecting their PCP. Some studies emphasized physicians’ proficiency being the most important factor that was prioritised by patients [27, 28]. However, in this study, more patients felt that physicians’ humanistic qualities and their physician-patient relationship played a greater role in their decision-making process. This may be due to the fact as that as most patients interviewed in this study were not medically trained, there is information asymmetry between physicians and patients, hence it may be difficult for them to determine the professional proficiency of physicians. As a result, patients would tend to make their decisions based on what they could evaluate, which would primarily be their interactions with the physicians as well as how the physicians carried themselves during the consultations.

The factors revealed in this study have been consistent with existing literature [21, 29]. The emphasis is on the partnership between the physician and patient [28, 30]. Patients valued a relationship with their PCPs underpinned by trust, which is also predictive of their satisfaction with their provider [31, 32]. Recurrent interactions with the same physician enables patients to develop trust in their healthcare provider [33]. Studies have also demonstrated a regular provider is associated with improved patient outcomes and satisfaction [34]. It is therefore important to ensure that the healthcare system embeds processes and measures to incentivize and sustain such long-term interpersonal relationships.

The findings summarize the key physician factors for PCPs to adopt and adapt to sustain their clinical practice when the healthcare system reverts to a capitation-based healthcare financing model. It is important for PCPs to have insight into patient preferences, so that their practices and patient interactions can be evolved to enhance the physician-patient relationship. One possible way would be to upskill PCPs in refresher communication and professionalism training programmes. Selected behavioural economics approaches and system changes can be applied to motivate the PCPs to sustain the care continuity of patients [21]. Other evidence-based interventions include changing default settings and publishing social reference points such as care qualities by the PCPs via official portals [35]. Reporting carefully selected clinical quality indicators among anonymized clinical practices may nudge the PCPs through “upward social comparison”. Pitching themselves to their peers can potentially motivate the PCPs to evolve to a more patient-centred clinical practice [36].

The Healthier SG has a national focus to promote strong relationships between the PCPs, irrespective of their clinical practices, and their patients in the local community [16]. At the individual level, the results allude to the readiness of patients to embrace changes under the Healthier-SG programme. PCPs in turn should also be adequately prepared and geared to provide care under the national programme.

The interviews in this study were conducted not only in English, but also in Chinese and Malay to ensure that voices across the local multi-ethnic residents were represented, and to capture the richness and nuances of their experience. Their perspectives were further widened through interviewing patients who consulted PCPs in public and private clinics.

This study has its limitations. The perspectives of PCPs are also crucial to maintain the continuity of care with their patients, which will be explored in a separate study. System-related factors may significantly impact decision-making among patients in their PCP selection. Due to the breadth and depth of the findings, the factors relating to the other domains depicted by the theoretical framework are reported in another publication. Generalizability is restricted due to the study method. A subsequent survey is planned to quantify and identify the major factors for an interventional trial to sustain the care continuity between PCPs and their patients.

## Conclusion

In a country with a dual primary healthcare model, patients with NCDs focused on physicians’ pleasant demeanour, empathy, and compassion as they selected their PCP. They valued PCPs who were competent and had effective interpersonal skills. Some of them selected their PCP based on compatible age and gender that they felt comfortable with. These factors contributed towards establishing good rapport with their PCP. Interventions to improve physician behaviour could be implemented to evolve their practices to deliver continuity of care to their patients.

## Supporting information

S1 File Topic guide. This is the semi-structured interview guide to investigate the factors affecting patient preferences in selecting a primary care provider.
(PDF)
