# Correction: Physician factors affecting patient preferences in selecting a primary care provider: A qualitative research study in Singapore

**DOI:** 10.1371/journal.pone.0330181

**Published:** 2025-08-11

**Authors:** Abigail Ern Jie Lee, Sulaiha Ithnin, Ngiap Chuan Tan

The second author’s name is spelled incorrectly. The correct name is: Sulaiha Ithnin. The correct citation is: Lee AEJ, Ithnin S, Tan NC (2024) Physician factors affecting patient preferences in selecting a primary care provider: A qualitative research study in Singapore. PLoS ONE 19(3): e0298823. https://doi.org/10.1371/journal.pone.0298823
