# Supplementary material for: Physician factors affecting patient preferences in selecting a primary care provider: A qualitative research study in Singapore
Source: PLoS One. 2024 Mar 1;19(3):e0298823. doi: 10.1371/journal.pone.0298823 (PMC10906824; doi:10.1371/journal.pone.0298823)
Supplement: S1 File — This is the semi-structured interview guide to investigate the factors affecting patient preferences in selecting a primary care provider. (PDF) [file pone.0298823.s001.pdf]

Physician factors affecting patient preferences in selecting a primary care provider: A qualitative research study in Singapore

Abigail Ern Jie Lee[1], Sulaiha Ithnin[1], Ngiap Chuan Tan[1,2]

[1]SingHealth Polyclinics, Singapore

[2]SingHealth-Duke NUS Family Medicine Academic Clinical Programme, Singapore

**Topic Guide**
You have seen doctor(s) for your health and medical conditions. We would like to seek your views on the care that you receive from various doctors in the community.<br><br>1. Please tell me more about your experience with your doctor recently.<br>2. Why do you choose to see that doctor?<br>3. Do you have choices in selecting the doctor? If yes, tell me more about how you choose the doctor.<br>4. Please share with me if there are other reasons for choosing the doctor, aside from the doctor himself/ herself?<br>5. Tell me more about the kind of doctor which will best take care of your health and medical condition.<br>6. Please share with me if your current doctor fits this description.<br>7. How would you feel if you are cared for by a regular team of doctors and nurses instead of one specific doctor?
